# Adipose tissue: a neglected organ in the response to severe trauma?

**DOI:** 10.1007/s00018-022-04234-0

**Published:** 2022-03-26

**Authors:** Lisa Wrba, Rebecca Halbgebauer, Julian Roos, Markus Huber-Lang, Pamela Fischer-Posovszky

**Affiliations:** 1 Institute of Clinical and Experimental Trauma Immunology, Ulm University Medical Center, Ulm, Germany; 2 Department of Trauma, Orthopedic, Plastic and Hand Surgery, University Hospital of Augsburg, Augsburg, Germany; 3 Department of Pediatrics and Adolescent Medicine, Ulm University Medical Center, Eythstr. 24, 89075 Ulm, Germany

**Keywords:** Adipose tissue, Adipokine, Trauma, Immune response

## Abstract

Despite the manifold recent efforts to improve patient outcomes, trauma still is a clinical and socioeconomical issue of major relevance especially in younger people. The systemic immune reaction after severe injury is characterized by a strong pro- and anti-inflammatory response. Besides its functions as energy storage depot and organ-protective cushion, adipose tissue regulates vital processes via its secretion products. However, there is little awareness of the important role of adipose tissue in regulating the posttraumatic inflammatory response. In this review, we delineate the local and systemic role of adipose tissue in trauma and outline different aspects of adipose tissue as an immunologically active modifier of inflammation and as an immune target of injured remote organs after severe trauma.

## Introduction: relevance of trauma and obesity

Severe trauma is still a major cause of disability and death. In 2017, 4.5 million people around the world died due to traumatic injuries. Trauma accounts for 8.0% of all deaths, and plays an even greater role in deaths of young people. Males face an almost twice as high death rate from injury compared to females. Additionally, there were 521 million of non-fatal injuries in 2017 [40]. Thus, despite improvements of clinical trauma management and increasing survival rates, trauma remains an important cause of adverse outcome and death.

Traumatic injuries induce a very complex systemic immune reaction, involving various parts of innate and adaptive immunity, changes in endothelial function, the coagulation system and endocrine signaling as reviewed in detail in [46]. Trauma causes systemic release of damage-associated molecular patterns (DAMPs) from damaged cells, and may also cause exposure to pathogen-associated molecular patterns (PAMPs) from the environment or the interior of the body (especially the gut). DAMPs and PAMPs activate immune cells, which increase their phagocytic capacity, secrete reactive oxygen species (ROS) and a variety of pro- and also anti-inflammatory cytokines. Interleukins (IL) like IL-6, IL-8, IL-10, tumor necrosis factor (TNF) and chemoattractants as monocyte chemoattractant factor 1 (MCP-1) are only some examples for mediators in the so-called cytokine storm provoked by severe injury. Furthermore, the complement system, involved in not only phagocytosis, but also immune modulation, is activated. The coagulation system is activated in order to stop harmful bleeding, with the risk of clot formation and hypoperfusion of tissue, aggravating systemic inflammation. Whether all those mechanisms are protective or harmful is very much dependent on the balance between pro- and anti-inflammatory mediators, and on the proportion of activators and inhibitors; an excessive activation of the mainly innate proinflammatory response may thus cause systemic inflammatory response syndrome (SIRS) and subsequent (multiple) organ failure [46].

Overweight and obesity have reached pandemic proportions. According to the World Health Organization (WHO), 1.9 billion adult people were overweight (BMI ≥ 25 kg/m^2^) and 650 million were obese (BMI ≥ 30 kg/m^2^) in 2016, corresponding to 39% or 13% of the global adult population [132]. The excessive accumulation of adipose tissue can lead to severe comorbidities, among them type 2 diabetes mellitus and cardiovascular diseases but also certain types of cancer. Of note, adipose tissue itself via its secretion products is involved in the pathogenesis [69].

In the case of a traumatic injury, adipose tissue is essential for the absorption of the inflicting trauma vector [9, 55, 103, 113]. Interestingly, an obesity paradox has been described in trauma patients: in this setting, a protective function of overweight has been described on overall survival, whereas obesity grade 2 and 3 (BMI ≥ 35 kg/m^2^) as well as underweight increased the risk of death after trauma [26]. Due to greater forces at lower speed, obese patients tend to suffer from more severe but more distally located fracture types caused by low-impact trauma [3, 5, 68, 97, 116]. In addition, the risk for impaired wound and fracture healing are higher in the obese population [3, 113]. This argues for an involvement of adipose tissue in the systemic response to trauma. However, its role on the immuno-pathophysiology, outcome, and treatment options after severe trauma remains rather undefined. Herein, we aim to examine and reflect the role of the adipocyte-immune-organ crosstalk in severely injured patients.

## Adipose tissue: more than just an energy store

Based on morphological features, adipose tissue can be roughly classified into two basic types, white adipose tissue (WAT), where univacuolar, white adipocytes are found, and brown adipose tissue (BAT), consisting of multivacuolar, mitochondria-rich, brown adipocytes [95].

WAT covers the body in the subcutaneous depot underneath the skin, while the inner viscera are surrounded by visceral depots. As fat is a poor conductor of heat, it insulates the body and protects against cooling, but it also provides support and protection. In healthy subjects, WAT accounts for approximately 20–25% of total body mass, but in obese subjects, it can increase to up to 50% [94]. WAT was long considered a passive organ where excess energy is stored in the form of triglycerides. Usually, lipids are ingested with food, and the uptake and storage of lipids is mainly regulated by the anabolic hormone insulin. In times of negative energy balance, e.g. during a period of fasting, fatty acids can be mobilized from the lipid droplets through the process of lipolysis [13]. Besides these vital metabolic functions, WAT has been increasingly recognized as important endocrine organ releasing a plethora of secretion products to the circulation, which have been named adipokines [95]. Among them are protein factors, e.g. classical hormones such as leptin or adiponectin, growth factors, chemokines and cytokines, but also certain lipid mediators, e.g. prostaglandins, and metabolites, e.g. fatty acids, nucleotides, nucleosides or lactate [141]. Via these adipokines, WAT is in continuous crosstalk with both the local microenvironment as well as all other organs systems in the body and regulates vital processes or functions such as food intake, energy homeostasis, insulin sensitivity, hemostasis, and blood pressure [31].

While WAT is best known for storing fat, BAT has the unique ability to use it for heat production in a process called non-shivering thermogenesis. The thermogenic function is achieved by uncoupling protein-1 (UCP1), which is located at the inner mitochondrial membrane. Upon activation, UCP1 uncouples the proton gradient, which is generated in the respiratory chain, from adenosine triphosphate (ATP) synthesis to produce heat instead of ATP. BAT can be found in the interscapular and paravertebral region and also in perirenal depots. For decades, it was believed that BAT in humans is only present in neonates where it is responsible for the maintenance of body temperature, but it is now well accepted that functional BAT is also present in adults. Interestingly, an intermediate beige (brite, inducible BAT-like) adipose tissue phenotype can emerge from WAT upon chronic cold exposure in a process referred to as browning [52]. Beige adipocytes can store triglycerides, but also express UCP1 and can therefore contribute to heat production. Of note, browning of WAT has recently been linked to the hypermetabolic response after burn injury, which will be discussed in detail below [54]. In addition to white, brown, and beige adipose tissue we would also like to mention an additional adipose tissue depot with potential relevance to trauma, i.e. marrow adipose tissue.

## Fat tissue as traumatized organ

As WAT is the major component of the subcutis, injury of adipose tissue is caused by nearly every physical trauma mechanism. Trauma generally induces a disruption of macro-barriers such as the skin and underlying soft tissue, as well as micro-barriers such as cell membranes, resulting in a complex immune response [46]. Damaged tissue releases various damage-associated molecular patterns (DAMPs), e.g., high-mobility group box 1 protein (HMGB1), ATP, nuclear contents such as histones, and mitochondrial DNA [137, 138, 140]. In adipose tissue, besides a necrosis-caused release of HMGB1, also an active secretion of this DAMP has been shown, especially in presence of inflammatory stimuli [38, 107, 137]. Of note, there is only limited data concerning the impact of blunt or penetrating trauma on WAT. In hematoma caused by fracture and concomitant soft-tissue injury, high concentrations of cytokines such as IL-6, IL-8 and TNF were detected [43]. Operative trauma or even minimal tissue injury caused by cannulas also increased the concentrations of inflammatory mediators [37, 79]. Burn injury in particular increased the expression of IL-6, IL-8, MCP-1, and TNF in WAT, but not of adipogenic, chondrogenic, or osteogenic factors [85, 98], and leads to morphological and functional changes, such as smaller adipocytes, collagen accumulation and tissue fibrosis, or invasion of macrophages and upregulation of UCP1. UCP1 is the key marker of brown and beige adipose tissue and characterized by its thermogenic capacity as discussed earlier. In this regard, increased expression of UCP1 was proposed to significantly contribute to the hypermetabolic state after burn trauma [1, 54, 85, 98]. Destruction of adipose tissue as well as endocrine mechanisms, e. g., β-adrenergic signaling, liberate fatty acids such as oleate and linoleate into the systemic circulation [7, 114, 126]. In case of penetrating trauma, invading bacteria with their PAMPs may exacerbate the inflammatory reaction by recruitment of further immune cells. In turn, these inflammatory cells, which represent neutrophils and monocytes/macrophages and therefore the “first cellular line of defense” can further secrete cytokines and chemokines, generate reactive oxygen species (ROS) and inflammatory mediators, with local effects as described below in detail [46, 53, 54].

## Traumatized adipose tissue as a modulator of the systemic post-traumatic response

WAT in different body compartments might react differentially to traumatic insults. Compared to subcutaneous adipocytes, visceral adipocytes are metabolically more active and have a higher capacity to generate free fatty acids (FFA) [47]. Visceral WAT contains larger numbers of immune cells and directly drains to the liver via the portal vein [47]. Although visceral adiposity was not associated with increased signs of inflammation [18], larger abdominal depots increased the risk of acute kidney injury [105].

**Fig. 1:**
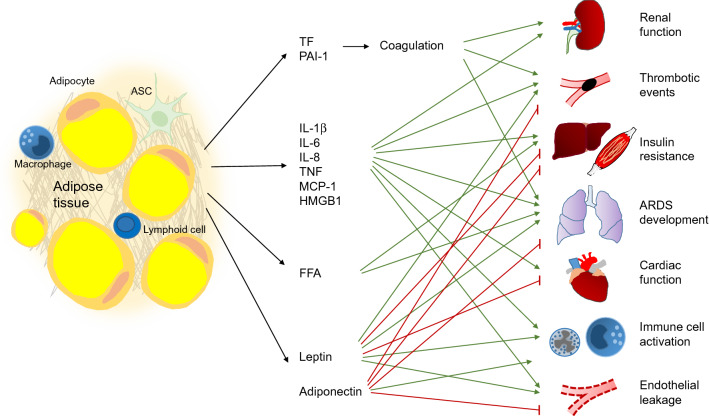
Adipose tissue as a modulator of systemic post-traumatic responses. After a severe traumatic insult, a plethora of factors is released into systemic circulation, many of which are also secreted by adipose tissue. Traumatized adipose tissue releases procoagulant mediators such as tissue factor (TF) and plasminogen activator inhibitor 1 (PAI-1), facilitating thrombotic events. Furthermore, various proinflammatory mediators such as interleukin (IL-) 1β, IL-6, IL-8, tumor necrosis factor (TNF), monocyte chemoattractant protein-1 (MCP-1) and high mobility group box 1 protein (HMGB1) are liberated. Together with free fatty acids (FFA), they elicit various effects such as insulin resistance and immune cell activation, and compromise physiological functions of various remote organs. The adipokine adiponectin might exert systemic anti-inflammatory effects. Leptin was recently described as a prognostic marker of multiple organ failure in critically ill patients. *ARDS* acute respiratory distress syndrome, *ASC* adipose stromal cell

Adipose tissue is an important source of inflammatory mediators such as cytokines (e.g., IL-1β, IL-6) and chemoattractants (e.g., MCP-1). Nevertheless, most of these mediators can also originate from other tissues and the exact impact of visceral and subcutaneous adipose tissue is still rather unclear. Potential mechanisms caused or influenced by mediators secreted from adipose tissue are described in this section and also shown in Fig. 1.

DAMPs and cytokines recruit inflammatory cells to the adipose tissue, further exacerbating release of cytokines [1, 54, 115]. These mediators exhibit various systemic effects on remote organs and are of prognostic value in patients after severe trauma, with higher concentrations associated with poorer outcome [20, 35, 63, 81]. Especially HMGB1, a DAMP passively liberated from adipose tissue and other tissues via cell death as well as actively through secretion processes, plays a pivotal role in remote organ injury by triggering the release of other cytokines such as IL-6, IL-10, and MCP-1 [38, 63, 107]. In addition, stressed adipocytes are able to enhance the protease release and oxidative burst reaction of neutrophils [23]. Inflammatory mediators, proteases, and ROS released into systemic circulation provoke remote organ injury by barrier disruption, protein degradation, cell death, and accumulation of inflammatory cells in remote organs [12, 46, 99]. The lungs are particularly susceptible to injury caused by distant organ trauma [12, 25, 90]. Severe soft (adipose) tissue trauma can be sufficient to robustly increase the numbers of inflammatory cells and especially neutrophils in the lungs and to disturb lung tissue oxygenation [39, 110]. These inflammatory mediators also contribute to renal failure [16, 76, 124] and can suppress cardiac function [67, 135] (Fig. 1). Certain adipokines such as IL-17A and leptin were recently described as potential diagnostic parameters in severely injured patients: their plasma concentrations were significantly elevated after severe multiple injury, with higher values associated with an increased risk of multiple organ failure (MOF) development [42]. For leptin, however, there is conflicting data. Some studies showed higher systemic concentrations after severe trauma [42, 80], while others detected reduced leptin levels in severely burned subjects [124] and critically ill patients after surgery [87]. Thus, further studies are required to determine the exact role of leptin in traumatized patients. Adiponectin is another important adipokine with anti-inflammatory features and protective systemic effects [8, 27, 120, 134], although there is some conflicting data in the context of critically ill patients [22]. Resistin and visfatin, two proinflammatory adipokines, were increased after burn injury and critical illness and associated with poorer outcome [4, 124]. However, to date, there is a lack of causative studies demonstrating the specific influence of these adipokines in the condition of severe trauma.

As another important aspect, higher BMI and increased fat mass alter the coagulation state after trauma towards a procoagulant phenotype and increase the risk for thrombosis and pulmonary embolism [77, 104, 128]. Alterations in blood thrombo-elastometric properties of obese subjects were shown in several studies, but the underlying mechanisms are not completely unraveled [57, 73, 128]. One possible factor is the production of plasminogen activator inhibitor 1 (PAI-1) in adipose tissue. As it possesses procoagulant features and its expression is enhanced by inflammatory mediators, PAI-1 might play an important role in posttraumatic thromboembolic events [73, 108, 121]. Moreover, WAT serves as an important site of synthesis of tissue factor (TF), which is a major activator of the coagulation cascade (Fig. 1). Therefore, liberation of TF from WAT after trauma may promote a procoagulant state [29]. IL-6 derived from WAT after trauma may furthermore induce synthesis of factor VII, factor VIII, and fibrinogen in the liver, further driving clot formation [29, 33].

Adipose tissue trauma and exposure to DAMPs and PAMPs as well as catecholamines (via β2 signaling) can liberate FFA into systemic circulation [82, 119]. FFA function as an important source of energy in a catabolic state, such as after severe injuries [126, 129]. On the other hand, FFA in mesenteric lymph and plasma have cytotoxic effects, for example on endothelial cells, thus promoting vascular leakage [15, 86, 112]. Released into systemic circulation, FFA can exhibit inflammatory features [7, 11]. Insulin resistance as a frequent condition in posttraumatic metabolism can also be promoted by FFA and other lipid degradation products such as diacylglycerols [21, 58, 101]. Recent studies showed a causal link between increased lipolysis, systemic insulin resistance and further metabolic disturbances, indicating a direct impact of metabolic changes in adipose tissue on the systemic posttraumatic reaction [54, 88]. Furthermore, elevated plasma concentrations of FFA were described as a risk factor for development of acute respiratory distress syndrome (ARDS) after trauma and sepsis [7] (Fig. 1).

Taken together, adipose tissue might act as an important player in the posttraumatic systemic response and contribute to remote organ damage and failure.

## Adipose tissue as a target of the systemic response after trauma

Severe trauma activates the sympathetic nervous system and thereby increases systemic levels of catecholamines such as epinephrine and norepinephrine, which positively correlate with the injury severity. High catecholamine levels are furthermore associated with poor prognosis [51, 92, 131]. Catecholamines have various effects on different tissues, such as the endothelium, muscles, and various organs [32, 50, 61, 70]. As trauma victims, especially in the case of an additional hemorrhagic shock, often suffer from early hypotension, external catecholamine administration is often required to restore and maintain a blood pressure sufficient for organ perfusion [127]. However, the metabolic state of adipose tissue is significantly altered by catecholamines. Lipolysis is stimulated via β-adrenergic pathways, liberating FFA as energy source for remote organs [93]. Excessive catecholamine stimulation may also result in a prolonged lipolytic activity with wasting of adipose tissue [14, 49]. IL-6 secreted by various tissues throughout the body after trauma also promotes lipolysis in adipocytes and decreases adipocyte size [41, 122]. As another inflammatory cytokine, TNF is able to induce lipolysis in adipose tissue and furthermore impairs its insulin sensitivity and lipid storage capacity [10].

WAT is able to change its phenotype in response to trauma and turn into beige adipose tissue with thermogenic capacity. Whether this occurs through transdifferentiation of white adipocytes or de novo differentiation of specific precursor cells is still a matter of debate [44]. The expression of UCP1, the key thermogenic factor, is induced after trauma and burn injury, not only at the injured site, but also in distant areas [2, 66, 109]. Browning of WAT naturally occurs under chronic cold exposure, but it can also be induced by inflammatory mediators such as IL-6, high circulating levels of catecholamines, or their local production by alternatively activated macrophages [1, 2, 34, 54], all playing key roles in the pathophysiology after severe trauma. Thus, it is intuitive that WAT browning was shown after burn injury, major surgical trauma, and conditions associated with high catecholamine concentrations [34, 66, 109]. In turn, adipose tissue browning induces a hypermetabolic state with reduction of storage depots, lower fat content, lower weight, decreased bone mineral content and increased levels of cortisol, catecholamines, cytokines, and acute phase proteins resulting in higher heart rate and cardiac output [49].

WAT and its residing immune cells produce cytokines and chemokines that can spill over into systemic circulation, but of course, it will also receive inflammatory signals from distant organs, further aggravating the inflammatory state in a vicious cycle. The inflammatory response of WAT is furthermore determined by the general health state of the patients and their fat mass. Importantly, it can be influenced by the preoperative diet, with dietary restriction reversing the detrimental effects of diet-induced obesity on the WAT inflammatory response to trauma, opening up therapeutic options [71, 78].

**Fig. 2:**
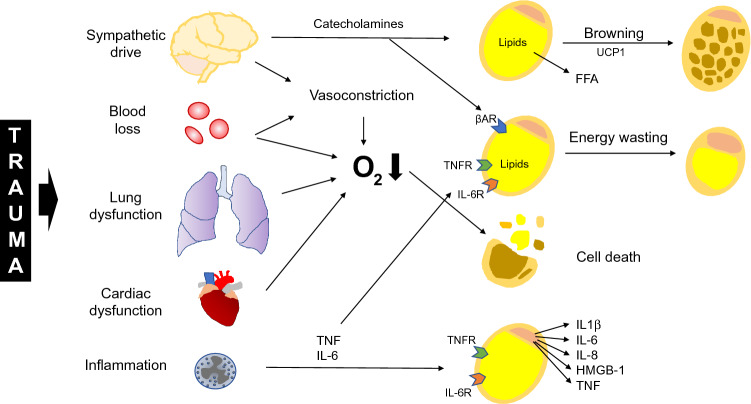
Adipose tissue as a target of post-traumatic systemic pathophysiological conditions. Severe trauma results in organ dysfunction, blood loss and a sympathetic reaction. The liberated catecholamines induce browning of white adipose tissue by upregulating expression of uncoupling protein 1 (UCP1). Thereby, heat is produced and free fatty acids (FFA) are liberated. Catecholamines (via β-receptors, βAR) as well as cytokines, e.g. tumor necrosis factor (TNF) and interleukin (IL) 6, induce lipolysis and energy wasting. Adipocytes are activated by proinflammatory mediators and secrete cytokines, aggravating inflammatory response. Furthermore, hypoxia caused by blood loss and centralization leads to cell death in adipose tissues

To summarize, catecholamines and inflammatory mediators dysregulate homeostasis of adipose tissue, resulting not only in a hypermetabolic state with reduction of the energy storage depots, but also in aggravation of the systemic inflammatory response by the adipose tissue itself (Fig. 2).

## Micromilieu changes in adipose tissue after trauma

**Fig. 3:**
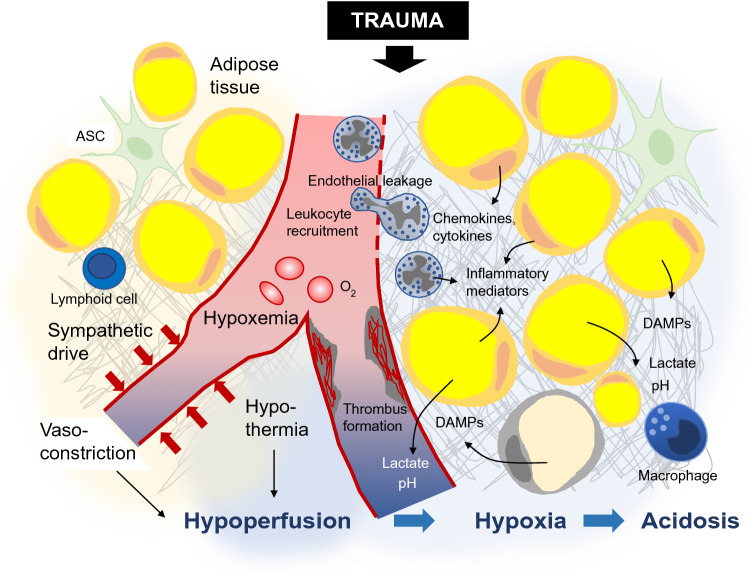
Changes of the micro-milieu at the blood-fat-tissue barrier after trauma. Severe trauma leads to barrier dysfunction, resulting in endothelial leakage, leucocyte recruitment and extravasation, and tissue inflammation. Tissue inflammation is further aggravated by adipocytes and local immune cells producing proinflammatory mediators. Some of these are again liberated into the circulation. Hypoxia, caused by blood loss, vasoconstriction and thrombus formation, promotes anaerobic metabolic pathways with generation of lactate. Thus, the posttraumatic micromilieu in adipose tissue is characterized by hypoperfusion, hypoxia, acidosis, hypothermia and a pronounced inflammatory reaction. *ASC* adipose stromal cell

Trauma alters the micromilieu in most tissues of the body and also in WAT. The so-called “lethal triad”, composed of hypothermia, acidosis and coagulopathy, is, although adapted during the past years, still of great value considering prognosis and therapeutic concepts in severely injured patients [24, 74, 106]. The lethal triad is a systemic concept, but the underlying components and mechanisms are also present in the micromilieu of adipose tissue. Due to its location at the body surface, subcutaneous WAT is particularly affected by hypothermia, as ambient temperature is usually lower than the physiological tissue temperature. Major trauma can cause substantial blood loss, resulting in hypoperfusion of tissues. Perfusion of non-vital tissues such as adipose tissue is furthermore compromised by vasoconstriction caused by endogenous catecholamines (mainly via β-adrenergic receptors) released in trauma and shock [46]. Hypoperfusion impairs removal of metabolic products and cell debris and plays a major role in the development of acidosis. With less oxygen available, anaerobic metabolic pathways leading to generation of acidic metabolites such as lactic acid become more important, further promoting adipose tissue acidosis [100, 118] (Fig. 3).

Acute trauma-induced coagulopathy is a complex process leading to formation of microthrombi, thereby worsening tissue perfusion. Furthermore, consumption of platelets, coagulation factors and other mediators contribute to traumatic coagulopathy development. Thus, posttraumatic coagulopathic states may result in a hemorrhagic phenotype aggravating injury-induced blood loss [17, 111]. As described above, adipose tissue plays an important role in development of trauma-induced coagulopathy, thereby hampering its own blood supply and hemostatic capacity [29, 73]. Endothelial leakage after trauma occurs in the vasculature of the whole body, increasing inflammatory cell invasion into tissues. As adipose tissue secretes various inflammatory cytokines and DAMPs as described above, it recruits inflammatory cells, thus further exacerbating tissue inflammation [1, 54, 115]. Inflammatory cells liberate ROS, again aggravating endothelial leakage and tissue acidosis, resulting in a vicious cycle of micromilieu changes [46]. Overall, these changes can cause blood-organ-barrier dysfunction and failure [46] and, in case of adipose tissue, a proposed “blood-adipose tissue barrier” dysfunction (Fig. 3).

## Adipocyte dysfunction after trauma

**Fig. 4:**
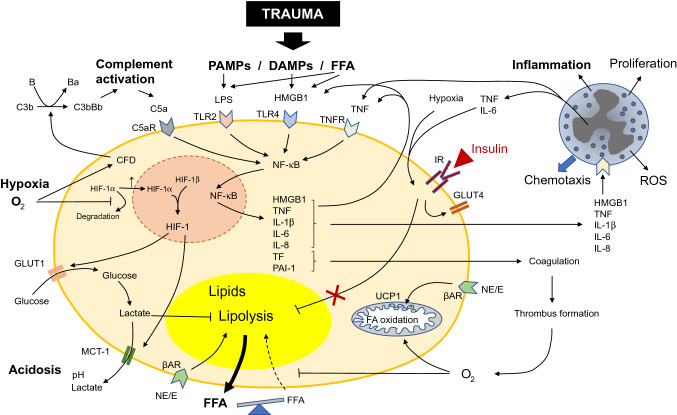
Posttraumatic adipocyte dysfunction. After trauma, adipocytes are exposed to pathogen-associated molecular patterns (PAMPs) and damage-associated molecular patterns (DAMPs), released locally and from neighboring damaged tissues. DAMPs, PAMPs and free fatty acids (FFA) activate receptors on the adipocyte surface, such as toll-like receptors (TLR). Thus, proinflammatory pathways as the NF-κB signaling pathway are upregulated, resulting in production and secretion of proinflammatory interleukins (IL), high mobility group box 1 protein (HMGB1), tumor necrosis factor (TNF) and procoagulant factors such as tissue factor (TF) and plasminogen activator inhibitor-1 (PAI-1). These cytokines activate immune cells, aggravating the inflammatory response. The procoagulant state facilitates thrombus formation, worsening hypoxic conditions. Thus, aerobic pathways (e.g. fatty acid (FA) oxidation) are downregulated, whereas anaerobic pathways such as glycolysis are upregulated, resulting in the generation of lactate, which is shuttled outside the cell via monocarboxylate transporter 1 (MCT-1), resulting in tissue acidosis. Hypoxia activates the hypoxia-induced 1 factor (HIF-1) pathway, upregulating glucose uptake via glucose transporter (GLUT) 1, and lactate release. Lipolysis is induced by epinephrine (E) and norepinephrine (NE) via β-receptors (βAR). After trauma, insulin sensitivity is impaired, further enhancing lipolysis and liberating FFA. By releasing complement factor D (CFD), adipocytes also activate the complement system, strengthening the posttraumatic response

Severe injuries result in adipose tissue hypoxia, as oxygen supply is reduced due to blood loss and vasoconstriction. Adipocytes react to hypoxic conditions by changing their metabolic pathways and endocrine functions. Hypoxic adipocytes up-regulate the glycolytic pathway in response to low oxygen supply, representing a shift towards anaerobic metabolism [36, 72]. Hypoxia-inducible factor 1 (HIF-1) has been identified as a major regulator of the metabolic shift in hypoxic cells. HIF-1 is a heterodimer with the β-subunit constitutively expressed independent of oxygen supply, while the α-subunit is rapidly degraded by the proteasomal complex under normoxic, but not under hypoxic conditions [102]. HIF-1 regulates various genes involved in glucose uptake and metabolism, angiogenesis, remodeling of extracellular matrix, apoptosis and inflammation [118]. Glucose transporter 1 (GLUT1) is one of the key targets upregulated via the HIF-1 pathway, resulting in increased glucose uptake of adipocytes. Gene expression, but not protein levels, of GLUT3 and GLUT5 has also been shown to increase in response to hypoxia, while expression of the insulin-sensitive transporter GLUT4 is diminished under hypoxic conditions, impairing insulin sensitivity of adipocytes [89, 118, 130]. Glucose is degraded via anaerobic glycolysis, resulting in increased lactate levels in adipocytes [83] (Fig. 4). HIF-2, which is also stabilized and activated under hypoxic conditions, may counteract the HIF-1-induced insulin resistance to some extent, as it was shown to improve insulin sensitivity of different cell types [59, 62, 91, 117].

To remove lactate from adipocytes, monocarboxylate transporter 1 (MCT-1) is upregulated by the transcription factor HIF-1. Lactate is thus shifted out of the cells towards the extracellular matrix and systemic circulation, promoting lactic acidosis [83]. Lactate again is an important regulator of adipocyte metabolism, as it diminishes lipolysis [65]. Independent of lactate signaling, there is also data suggesting increased lipolytic activity in hypoxic adipocytes and reduced uptake of FFA, with higher systemic FFA concentrations [136]. Insulin sensitivity of adipocytes can be altered by different mechanisms after trauma. First of all, hypoxia directly impairs insulin signaling in a HIF-1-dependent manner [89, 136]. Furthermore, inflammatory mediators such as IL-6 and TNF can further promote insulin resistance in adipocytes [45, 60, 96].

An inflammatory reaction of adipocytes can be activated by sensing of DAMPs and PAMPs, but also of saturated FFAs by classical toll-like receptors (TLRs) such as TLR2 and TLR4 [28, 56] (Fig. 4). In severe trauma, PAMPs and DAMPs can originate from distant tissues and various cell types, but also adipocytes themselves produce DAMPs as HMGB1 and release FFAs, amplifying the local inflammatory response and activating immune cells [38, 119]. These effects are at least partly mediated via the NF-κB pathway. This key pathway can be activated by inflammatory cytokines, complement activation factors (e.g. C5a), TNF, or PAMPs (e.g. lipopolysaccharides, LPS), and promotes adipocyte metabolic dysfunction and secretion of inflammatory mediators [6, 139]. HIF-1, activated by low oxygen supply, is not only a key mediator of metabolic processes but also promotes the secretion of IL-6, PAI-1, vascular endothelial growth factor (VEGF) and various other factors [125], creating an autocrine and paracrine cycle of stimulation, further aggravating the local inflammatory response [10, 28]. Inflammation is further promoted by recruited immune cells (e.g. neutrophils) which are locally activated by adipocyte-derived products [23]. This principle is also true for the fluid phase of the innate immune response mainly mounted by the complement system. For example, hypoxic adipocytes increase the generation and secretion of complement factor D (adipsin), which promotes activation of the alternative complement pathway [123, 125] (Fig. 4). As complement activation plays an important role in the pathophysiology after severe trauma, this displays another link between adipocytes and the immune response [46].

In conclusion, trauma alters metabolic functions of single adipocytes, affecting glucose utilization and both local and systemic insulin sensitivity [19, 64], causes WAT tissue browning, and results in release of FFAs and inflammatory mediators, all of which in concert lead to local (tissue) and systemic (blood) inflammation and disturbance of the metabolic balance.

## Trauma and the obesity paradox

As mentioned earlier, overweight can reduce mortality in severely injured patients, whereas severe obesity (BMI ≥ 35 kg/m^2^) and underweight increase the mortality risk [26]. Several explanatory approaches were published in the past years. Overweight causes constant low-grade inflammation effecting the immune system as well as metabolism, but potentially increasing the systemic tolerance for the strong immune response following trauma. Furthermore, overweight causes increased systemic levels of leptin compared to lean individuals. The adipokine leptin acts as an important modulator of immune responses, as it influences T cell function and thereby protects against infections, but on the other hand reduces systemic susceptibility to the toxicity of proinflammatory stimuli [30]. In addition, overweight patients have greater energy reserve during the catabolic states after severe injury [26]. These protective factors might be outweighed by negative consequences if body mass is further increased. Obese patients suffer from pre-conditions such as decreased lung volume, impaired expiratory air flow, reduced lung compliance and reduced gas exchange, resulting in hypoventilation and hypoxia [48]. Severely obese patients required longer duration of mechanical ventilation, resulting in ventilator-associated complications as pneumonia. They were also at higher risk for ARDS and multiple organ failure. This might be due to the harmful effects of hypoxia and hypoperfusion on vital organs, and an increased inflammatory reaction associated with higher grade obesity [77]. Thus, reduced mortality in critically injured patients due to moderate overweight may be offset by the deleterious pre-existing conditions in severe obesity.

## Outlook

Based on the recent advantages in understanding the impact of adipose tissue in acute diseases such as during the acute and subacute phase following trauma, novel interventional approaches and new aspects of established therapeutics have emerged to prevent the augmentation and continuance of an excessive inflammatory response. In this regard, pharmacological intervention to inhibit lipolysis and β-adrenergic signaling [75] or to improve insulin sensitivity both systemically but also locally in WAT, e.g., by administration of a PDGF receptor tyrosine kinase inhibitor [84], have demonstrated positive effects in preclinical and some clinical studies of acute injury. Future preclinical studies, e.g., in mouse models with either absence or excessive accumulation of WAT as well as large-scale multi-layered analyses in traumatized patients as recently done by the PAMPer consort [133] may provide more insights into the function of adipose tissue in regulating the host response to trauma and ensuing SIRS. Furthermore, modulating the local and systemic immune response by blocking excessive amounts of central proinflammatory mediators may circumvent the detrimental alterations of the micro-milieu and resulting dysfunction of adipocytes, thereby restoring a balanced posttraumatic response in adipose tissue.

## Data Availability

This review article does not report primary data or materials.
